# Differences of Anteroposterior Pelvic Radiographs Between Supine Position and Standing Position in Patients with Developmental Dysplasia of the Hip

**DOI:** 10.1111/os.12574

**Published:** 2019-11-14

**Authors:** Guo‐yue Yang, Ya‐yue Li, Dian‐zhong Luo, Cheng Hui, Kai Xiao, Hong Zhang

**Affiliations:** ^1^ Department of Orthopaedic Third Central Hospital of Tianjin Tianjin China; ^2^ Tianjin Institute of Hepatobiliary Disease Tianjin China; ^3^ Tianjin Key Laboratory of Artificial Cell Tianjin China; ^4^ Artificial Cell Engineering Technology Research Center of Public Health Ministry Tianjin China; ^5^ The Fourth Medical Center of the Chinese People's Liberation Army General Hospital Department of Orthopaedic Beijing China

**Keywords:** Developmental dysplasia of the hip, Osteotomy, Pelvic tilt, Radiography, Shooting position

## Abstract

**Objective:**

To explore the difference in pelvic tilt and hip joint parameters with developmental dysplasia of the hip (DDH) comparing the anteroposterior (AP) pelvic radiographs taken in supine and standing positions.

**Methods:**

A prospective study of DDH patients undergoing Bernese periacetabular osteotomy (PAO) was conducted. AP pelvic radiographs were taken in supine and standing positions before surgery The pelvic tilt and hip joint parameters from the two radiographs were compared. Contrast parameters included the distance between the pubic symphysis to sacrococcygeal distance (PSSC), lateral center‐edge angle (LCEA), Tönnis angle (TA), and angle of sharp (SA).

**Results:**

A total of 110 young DDH patients were enrolled, including 32 men and 78 women, aged 18–49 years. The male PSSC was 45.63 ± 13.69 mm in supine position and 36.91 ± 12.33 mm in standing position (*P* < 0.05). The female PSSC was 56.76 ± 13.54 mm in supine position and 48.62 ± 15.44 mm in standing position (*P* < 0.05). In this study, LCEA <20° in AP pelvic radiographs in the supine position was found in 52 men and 135 women. For male patients, in supine position and standing position, LCEA were 5.51° ± 11.88° and 4.45° ± 12.22°, respectively (*P* < 0.05); TA were 20.20° ± 9.63° and 21.30° ± 9.97°, respectively (*P* < 0.05), and SA comparison showed no significant differences. For female patients, in supine position and standing position, LCEA were 3.07° ± 12.07° and 1.69° ± 12.11°, respectively (*P* < 0.05), TA were 22.62° ± 9.31° and 23.82° ± 9.45°, respectively (*P* < 0.05), and SA were 48.01° ± 4.68° and 48.49° ± 4.74°, respectively (*P* < 0.05).

**Conclusion:**

Compared with the supine position, the young DDH patients have pelvic tilt backward and a decrease in hip coverage in the standing position.

## Introduction

Anteroposterior (AP) pelvic radiographs are usually taken in supine position. In recent years, surgeons have tended to take AP pelvic radiographs in standing position as it obtains acetabular coverage and joint appositions in functional position^1^. Except that the pelvis needs to bear the weight of the upper body in standing position, the question remains of whether there are any other differences between the two positions. Konishi *et al*.^2^ measured the lateral pelvic radiographs and reported the presence of an approximate 5° difference in the pelvic tilt between supine position and standing position. Although lateral pelvic radiographs can be used to assess the pelvic tilt, there are particular requirements in regard to shooting position. Several shots were required to obtain a standard lateral pelvic radiograph. In addition, the overlap of bony structures also increases the difficulty of measurement, thus affecting the accuracy. Tannast *et al*.^3^ suggested that pubic symphysis to sacrococcygeal distance (PSSC) indirectly reflects the sagittal tilt of the pelvis by comparing the AP pelvic radiographs in supine and standing positions. This method has also been accepted and applied by other scholars^4^. Whether the differences in pelvic sagittal tilt between the two postures leads to corresponding changes in hip joint parameters remains controversial. Ross *et al*.^1^ investigated patients with femoral acetabular impingement (FAI) and found that lateral center‐edge angle (LCEA) demonstrated no significant differences in AP pelvis radiographs taken in supine and standing positions. Troelsen *et al*.^5^ measured the AP pelvic radiographs of 31 patients with developmental dysplasia of the hip (DDH) and reached a similar conclusion with LCEA, Tönnis angle (TA), hip joint space, and other hips in the AP pelvic radiographs taken in both supine and standing positions. The joint parameters did not demonstrate much change. While few other scholars hold different views, in studying cadaver specimens, Henebry *et al*.^6^ suggested that the sagittal tilt of the pelvis affects the hip joint parameters such as LCEA and TA. Pullen *et al*.^4^, in studying FAI, demonstrated significant differences in the acetabular parameters such as LCEA, TA, and angle of sharp (SA) in the AP pelvic radiographs taken in supine and standing positions.

Comparative studies of AP pelvic radiographs taken in supine and standing positions more focused on FAI^4^, ^6^, ^7^. There are few studies on DDH, and the study subjects have mainly included DDH patients with severe osteoarthritis. These patients are older, and the main treatment for them is total hip arthroplasty (THA)^5^. Young DDH patients have milder degrees of osteoarthritis, and periacetabular osteotomy (PAO) is often used to correct the deformities and slow down the progression of osteoarthritis. These patients have strong compensatory ability and have different spine–pelvis sagittal morphological characteristics when compared with elderly DDH and FAI patients. At present, there are no studies regarding the influence of different positions of radiographs on pelvic tilt and hip joint parameters in young DDH patients.

Hence, the present study aimed to determine whether the two different positions can cause changes in the pelvic tilt and whether the commonly used hip joint parameters, such as LCEA, TA, and SA, undergo significant changes when the two positions are changed by comparing the AP pelvic radiographs of young DDH patients in both supine and standing positions^5^, ^8^.

## Materials and Methods

This is a prospective study conducted on DDH patients undergoing PAO. With the permission of the ethics committee and after obtaining informed consent from the patient, AP pelvic radiographs in both supine and standing positions were taken before surgery. For this study, DDH patients had to meet the following criteria: (i) LCEA in AP pelvic radiographs in supine position should be less than 20° and of Hartofilakidis type I^9^; (ii) AP pelvic radiographs in supine and standing positions should be taken using a standard method, which can be accurately measured; (iii) Tönnis stage^10^ I and II of osteoarthritis; (iv) range of hip joint motion is within or beyond the normal range; and (v) the age of the patients should range from 18 to 50 years. Exclusion criteria were as follows: patients (i) with a history of spinal deformity; (ii) with a history of pelvic and lower limb fractures and related operations; (iii) with neurological diseases such as poliomyelitis and cerebral palsy; (iv) aged older than 18 years, epiphysis not closed; and (v) with a previous history of contralateral or ipsilateral hip joint surgery.

From October 2015 to April 2019, data for 118 patients with DDH who underwent PAO were collected, which included data for 8 patients with a previous history of hip joint and lower limb surgery. Finally, 110 cases were enrolled in this study, including 32 men and 78 women, aged 18–49 years, with an average age of 27.59 years. LCEA was measured in AP pelvic radiographs in supine position, and DDH was diagnosed when LCEA was less than 20°. Among the 110 cases, there were 27 cases with unilateral lesions, including 6 men and 21 women, and 83 cases with bilateral lesions, including 26 men and 57 women.

The GE Definium 6000DR X‐ray machine and the Unisight image processing system (GE Company USA) were used for shooting. It is necessary to keep both the lower limbs parallel, with both feet rotating 15°–20° internally, and to touch the toes of both feet when taking AP pelvic radiographs in the supine position. The subjects were asked to stand, rotate their legs in parallel, with both feet rotating internally, to touch their toes, and to distribute their body weight equally on their lower limbs as far as possible. The X‐ray tube was placed in the central position between the pubic symphysis and the bilateral anterior superior iliac spine, and the distance between the tube and the film was adjusted to 120 cm. The radiographs were saved in JPG format. Age, sex, height, and weight of the patients were recorded, numbered, and arranged in random order. Uniweb Viewer version 4.0 software was used to measure the relevant parameters.

For each patient, the AP pelvic radiographs were taken in two different positions and measured twice by two trained measurers; the interval between the two measurements was at least 4 weeks. The difference between different measurers and the difference between the two measurements by the same measurer were evaluated using a double blinding method.

**Figure 1:**
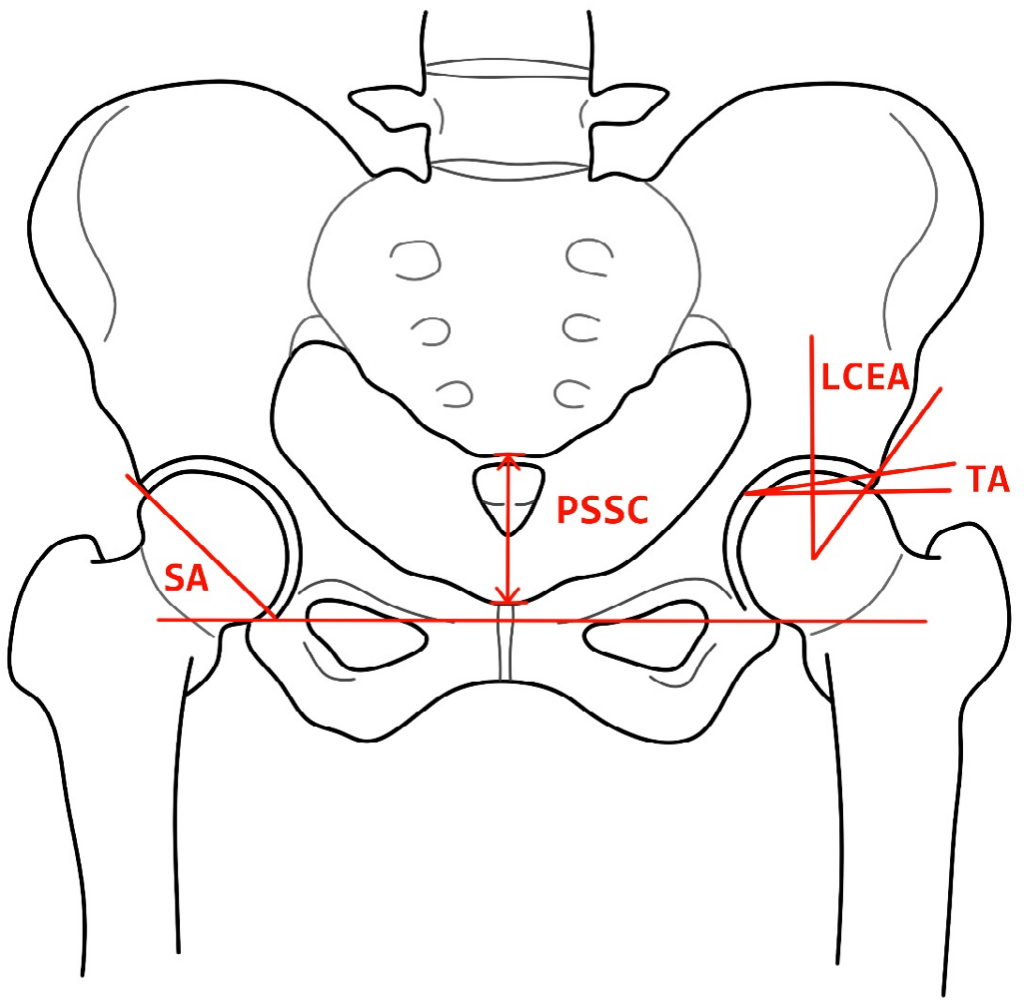
Anteroposterior (AP) pelvic radiograph in standing position is shown. PSSC is the distance between the upper edge of the pubic symphysis and the sacrococcygeal joint. LCEA and TA are the lateral center edge angle and Tönnis angle, respectively; SA is the sharp angle.

For all the measured angles, a horizontal reference between bilateral radiographic teardrops or a vertical reference between the midline pubic symphysis and sacrum was used^5^. PSSC was defined as the distance between the upper edge of the pubic symphysis and the sacrococcygeal joint (Fig. 1), reflecting the sagittal inclination of the pelvis^5^, ^11^. The PSSC of the same person has changed from large to small, indicating that the pelvis is tilted backward.

The LCEA was calculated by superimposing a circle over the acetabulum and measuring the angle between a vertical reference and the lateral edge of the sourcil, with the apex at the center of the superimposed circle. When the angle was less than 20°, DDH^12^ was diagnosed.

The TA was measured between a horizontal reference and a line formed parallel to the most medial and lateral extents of the sclerotic weight‐bearing portion of the acetabulum. The normal value was 0°–10° and more than 10° was considered DDH. The greater the angle was, the poorer the acetabular development was^10^.

The SA's apex was centered at the inferior radiographic teardrop and measured between a vertical reference and the lateral acetabular rim^13^ (Fig. 1). The SA has a reference value of approximately 45°. As the Sharp's angle becomes larger, hip coverage becomes worse.

### *Statistical Analysis*

Stata 9.2 software package (Stata, USA) was used for statistical analysis of the data. We compared the imaging parameters between supine and standing positions. PSSC, LCEA, TA, and SA corresponded to normal distribution and were evaluated by paired *t*‐test (quantity). Pearson correlation analysis was used to test the correlation between different measurements and the two measurements by the same measurer (quality). *P* < 0.05 (level of the test) was considered statistically significant.

## Results

### *Characteristics of Pelvic Tilt Changes in Patients with Developmental Dysplasia of the Hip from Supine to Standing Position*

From supine to standing position, PSSC was decreased. The male PSSC was 45.63 ± 13.69 mm in supine position and 36.91 ± 12.33 mm in standing position, showing significant differences (*P* < 0.05). The female PSSC was 56.76 ± 13.54 mm in supine position and 48.62 ± 15.44 mm in standing position, showing significant differences (*P* < 0.05, Table 1). PSSC in supine position was significantly different when compared with that in standing position.

**Table 1:** Pubic symphysis to sacrococcygeal distance (PSSC) in supine and standing position

Radiographic parameter		*n*	Supine	Standing	Difference	*P*
			($\bar{x}$ ± s)	($\bar{x}$ ± s)	($\bar{x}$ ± s)	
PSSC(mm)	Female	78	56.76 ± 13.54	48.62 ± 15.44	−8.13 ± 13.02	<0.000
	Male	32	45.63 ± 13.69	36.91 ± 12.33	−8.72 ± 10.80	<0.000

There were 57 female patients with bilateral onset and the difference in PSSC between supine position and standing position was −7.73 ± 12.91 mm, while there were 21 female patients with unilateral onset and the difference in PSSC between supine position and standing position was −9.23 ± 13.58 mm, showing no significant differences (*P* > 0.05). There were 26 male patients with bilateral onset and the difference in PSSC between supine position and standing position was −10.29 ± 11.30 mm, while there were 6 male patients with unilateral onset and the difference in PSSC between supine position and standing position was −1.58 ± 4.41 mm, with no significant differences (*P* > 0.05, Table 2) There was no significant difference in PSSC between supine position and standing position in patients with unilateral or bilateral DDH.

**Table 2:** Pubic symphysis to sacrococcygeal distance (PSSC) with unilateral and bilateral illness between supine and standing position

PSSC (mm)		*n*	Supine		Standing	Difference	*P*
Bilateral	Female	57	56.26 ± 14.21		48.53 ± 16.36	−7.73 ± 12.91	0.000
	Male	26	48.27 ± 13.47		37.98 ± 12.81	−10.29 ± 11.30	0.000
Unilateral	Female	21	58.11 ± 11.76		48.98 ± 12.98	−9.23 ± 13.58	0.005
	Male	6	34.42 ± 7.93		32.62 ± 9.66	−1.58 ± 4.41	0.419

From supine to standing position, PSSC showed great variability (with a range of −56.2–19.6 mm). The PSSC was decreased (pelvic posterior tilt) in 85 cases (59 women, 26 men) and increased (pelvic anterior tilt) in 25 cases (19 women, 6 men). From supine position to standing position, the pelvic posterior tilt (77.27%) was the main trend.

### *Characteristics of Hip Cover Changes from Supine to Standing Position in Patients with Developmental Dysplasia of the Hip*

**Figure 2:**
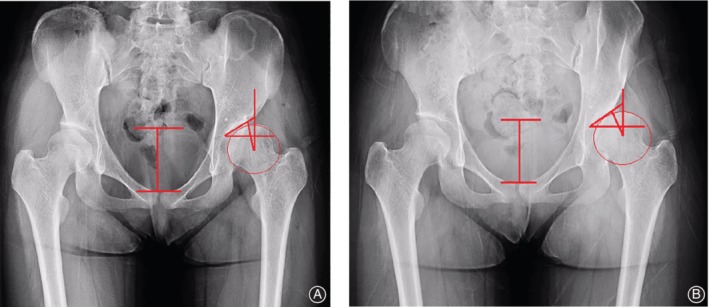
Anteroposterior (AP) pelvic radiograph is shown. (A) Pubic symphysis to sacrococcygeal distance (PSSC) is 6.36 mm, lateral center‐edge angle (LCEA) is −10.70°, and Tönnis angle (TA) is 35.20° in supine position; (B) PSSC is 5.94 mm, LCEA is −20.20°, and TA is 41.80°. From supine to standing, PSSC and LCEA decreased; TA increased.

**Figure 3:**
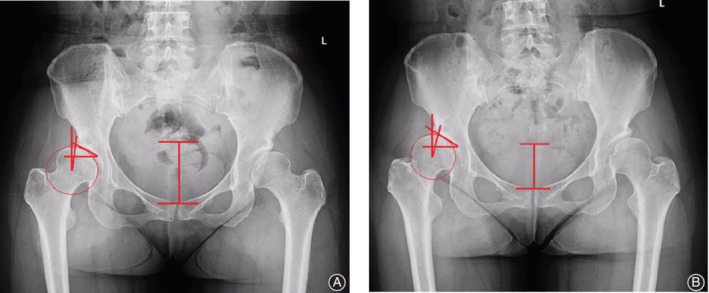
Anteroposterior (AP) pelvic radiograph is shown. (A) Pubic symphysis to sacrococcygeal distance (PSSC) is 5.96 mm, lateral center‐edge angle (LCEA) is −12.00°, and Tönnis angle (TA) is 30.87° in supine position; (B) PSSC is 4.94 mm, LCEA is −16.20°, and TA is 32.81° in standing position. From supine to standing, PSSC and LCEA decreased; TA increased.

In this study, LCEA <20° in AP pelvic radiographs in the supine position was found in 52 men and 135 women. For male patients, in supine position and standing position, LCEA were 5.51° ± 11.88° and 4.45° ± 12.22°, respectively (*P* < 0.05), TA were 20.20° ± 9.63° and 21.30° ± 9.97°, respectively (*P* < 0.05), and SA comparison showed no significant differences. For female patients, in supine position and standing position, LCEA were 3.07° ± 12.07° and 1.69° ± 12.11°, respectively (*P* < 0.05), TA were 22.62° ± 9.31° and 23.82° ± 9.45°, respectively (*P* < 0.05), and SA were 48.01° ± 4.68° and 48.49° ± 4.74°, respectively (*P* < 0.05). These results showed that LCEA was decreased, and TA and SA were increased in DDH patients from supine position to standing position (Table 3). (Figs 2 and 3).

**Table 3:** Acetabular measurements of the supine and standing positions

Radiographic parameter		*n*	Supine	Standing	Difference	*P*
*LCEA,deg*	Male	52	5.51 ± 11.88	4.45 ± 12.22	−1.06 ± 2.86	0.010
	Female	135	3.07 ± 12.07	1.69 ± 12.11	−1.37 ± 5.50	0.004
*TA,deg*	Male	52	20.20 ± 9.63	21.30 ± 9.97	1.10 ± 3.18	0.016
	Female	135	22.62 ± 9.31	23.82 ± 9.45	1.20 ± 4.03	0.001
*SA,deg*	Male	52	47.85 ± 5.43	47.97 ± 5.67	0.12 ± 3.46	0.0804
	Female	135	48.01 ± 4.68	48.49 ± 4.74	0.48 ± 2.65	0.035

The correlation coefficients of the same observer during different measurement periods were 0.72 and 0.92 (95% *CI*, 0.54–0.96), while the correlation coefficients of different observers were 0.69 and 0.96 (95% *CI*, 0.64–0.97).

## Discussion

Our study showed that from supine to standing position, pelvic posterior tilt was the main trend for DDH patients with Hartofilakidis type I and unilateral or bilateral onset was not considered as an independent factor for pelvic tilt. From supine position to standing position, LCEA was decreased and TA was increased, indicating that the hip joint coverage in standing position was worse.

### *The Trend Characteristics of Pelvic Sagittal Tilt in Young Developmental Dysplasia of the Hip Patients from Supine Position to Standing Position*

There are many methods to evaluate pelvic sagittal tilt in the published literature. It is easy to evaluate pelvic tilt indirectly by measuring PSSC on AP pelvic radiographs^2^, ^3^. Siebenrock *et al*.^13^ reported that the reference value of PSSC in supine position was 3.23 cm (2.5–4.0 cm) for male and 4.73 cm (4.0–5.5 cm) for female patients. Pullen *et al*.^4^ measured the PSSC in supine and standing positions in 46 FAI patients. The reference values were 55.8 mm (12–91.5 mm) in the supine position and 44.9 mm (24.5–78.6 mm) in the standing position for women, and 37.0 mm (14–64 mm) in the supine position and 20.6 mm (−10.7–47 mm) in the standing position for men. Tannast *et al*.^3^ reported no gender differences in the pelvic tilt between patients with FAI and DDH. The number of research samples in the above three studies was relatively small, which was not enough to fully reflect the weak difference between FAI and DDH. Kojima *et al*.^14^ compared the 3‐D morphological characteristics of the pelvis of normal people and DDH patients using CT. The results revealed that the pelvic morphology of DDH patients was different from that of normal people. Therefore, the results of FAI patients could not reflect the characteristics of pelvic tilt in DDH patients. In addition, it has been reported that the lumbar lordosis was decreased, and the pelvic posterior tilt was increased in the elderly when compared with young people^15^, ^16^, ^17^, ^18^. PAO was performed mainly in young DDH patients with mild hip osteoarthritis and stronger spinal and pelvic sagittal compensation. Theoretically, young DDH patients had different characteristics of pelvic tilt between FAI patients and elderly DDH patients.

Hip‐spine syndrome (HSS) was defined as the phenomenon of hip disease affecting the sagittal shape of the spine^19^. After the changes in spinal morphology, pelvic tilt is a compensation to achieve a new sagittal mechanical balance^15^, ^16^, ^17^, ^18^. In this study, PSSC was used to reflect the condition of pelvic tilt. From supine position to standing position, 77.27% of the patients had pelvic posterior tilt. Meanwhile, the mean value of PSSC was also decreased, indicating that the pelvic posterior tilt in standing position was the main trend. Without any doubt, nearly one‐quarter of patients showed pelvic anterior tilt from supine position to standing position, requiring individualized analysis of the characteristics of pelvic tilt. Especially for patients whose pelvic tilt changes were significant with body position, the influence of pelvic tilt changes should be fully considered when undergoing a PAO operation.

The degree of pelvic tilt in HSS patients is influenced by the degree of hip joint lesion and the compensation ability of the spine–pelvis in the sagittal plane. Matsuyama *et al*.^20^ hypothesized that patients with congenital dislocation of hip joints had severe pelvic anterior tilt, and the sacral slope could reach 68° on average. The body needed to compensate by increasing lumbar lordosis to retain sagittal balance. Different from the congenital dislocation of the hip joints, the DDH patients with Hartofilakidis type I are mainly characterized by shallow acetabulum and poor coverage, which had a relatively small effect on the sagittal shape of the spine–pelvis^21^. Whether unilateral or bilateral onset affects pelvic tilt in DDH patients has not been discussed in the published literature. In this study, we compared PSSC of unilateral and bilateral DDH patients, and found no significant difference between them. We believed that unilateral or bilateral onset of DDH patients with Hartofilakidis type I was not an independent factor for pelvic tilt.

### *Differences in Acetabular Parameters Between Supine and Standing Radiographs in Young Developmental Dysplasia of the Hip Patients*

At present, PAO is commonly used to delay the progression of osteoarthritis in young DDH patients, usually in supine position. However, the functional position of humans in daily life is standing. Therefore, we believed that in the preoperative design of PAO, the key to a successful operation is to adjust the rotation angle of the acetabulum in supine position and finally achieving a good coverage of the acetabulum in standing position. It is controversial whether the position of AP pelvic radiographs causes changes in hip joint parameters. Some scholars believe that the hip joint parameters are changed slightly from supine position to standing position, while others believe that shooting position might change the measurement results of acetabular coverage^13^, ^22^, ^23^. These studies are mainly based on the measurement and statistics of clinical imaging data. In studies on cadaveric models, some scholars report that the parameters of acetabular coverage would change with the change in pelvic tilt^4^, ^6^. In this study, the LCEA was decreased and TA was increased in young DDH patients from supine position to standing position, suggesting that the acetabular coverage in standing position was decreased. Although the average change of LCEA and TA was only 1°, the results were reasonable. The 1° change of LCEA and TA had little effect on the surgical results. However, it should be emphasized that the changes of LCEA and TA are related to the changes in pelvic tilt and the changes of hip joint parameters are more significant and should be considered in patients with high pelvic mobility. Uncertainty regarding the pelvic posterior tilt from supine position to standing position is the main trend. In addition, some patients have pelvic anterior tilt. Therefore, surgeons should fully evaluate the characteristics and trends of the pelvic tilt of each patient, adjust the rotation angle of the acetabulum, appropriately increase or decrease the rotation angle of the acetabulum osteotomy block when conducting preoperative planning to obtain good postoperative acetabulum coverage in the standing position, and realize the individualized design of the operation.

### *Limitations*

The AP pelvic radiographs were taken strictly in accordance with the shooting standards and the registration of radiographs was carried out through fixed length markers, which might still lead to systematic errors as a result of inspection methods. In addition, the small sample size of male patients cannot fully reflect the characteristics of the pelvis and hip joints of male DDH patients, causing certain systematic bias. Only a qualitative conclusion on the characteristics of pelvic tilt is presented in this study, and further quantitative studies are warranted. Although the changes between LCEA and TA in two positions were regular, the average changes were small, which might be questioned by surgeons. Because of the limitations of research methods, the parameters of anterior hip joint coverage were not included in this study.

### *Conclusion*

Most of the young DDH patients were with Hartofilakidis type I inclined pelvis backward from supine position to standing position. There was no significant difference in the degree of pelvic tilt between unilateral and bilateral DDH patients. From supine position to standing position, the acetabular coverage of DDH patients remained poorer. We recommend that AP pelvic radiographs should be taken in supine and standing positions before PAO, and the rotation angle of the acetabulum should be adjusted in the preoperative design to achieve more appropriate coverage of the hip joint in the functional position.
